# Shiga Toxin Uptake and Sequestration in Extracellular Vesicles Is Mediated by Its B-Subunit

**DOI:** 10.3390/toxins12070449

**Published:** 2020-07-10

**Authors:** Annie Willysson, Anne-lie Ståhl, Daniel Gillet, Julien Barbier, Jean-Christophe Cintrat, Valérie Chambon, Anne Billet, Ludger Johannes, Diana Karpman

**Affiliations:** 1 Department of Pediatrics, Clinical Sciences Lund, Lund University, 22185 Lund, Sweden; annie.willysson@med.lu.se (A.W.); anne-lie.stahl@med.lu.se (A.-l.S.); 2 Université Paris-Saclay, CEA, INRAE, Médicaments et Technologies pour la Santé (MTS), SIMoS, 91191 Gif-sur-Yvette, France; daniel.gillet@cea.fr (D.G.); julien.barbier@cea.fr (J.B.); jean-christophe.cintrat@cea.fr (J.-C.C.); 3 Institut Curie, PSL Research University, U1143 INSERM, UMR3666 CNRS, Cellular and Chemical Biology Unit, Paris, France; Valerie.Chambon@curie.fr (V.C.); anne.billet@curie.fr (A.B.); Ludger.Johannes@curie.fr (L.J.); 4 Université de Paris, F75005 Paris, France

**Keywords:** Shiga toxin, extracellular vesicles, red blood cells, HeLa cells, globotriaosylceramide

## Abstract

Shiga toxin (Stx)-stimulated blood cells shed extracellular vesicles (EVs) which can transfer the toxin to the kidneys and lead to hemolytic uremic syndrome. The toxin can be taken up by renal cells within EVs wherein the toxin is released, ultimately leading to cell death. The mechanism by which Stx is taken up, translocated, and sequestered in EVs was addressed in this study utilizing the B-subunit that binds to the globotriaosylceramide (Gb3) receptor. We found that Stx1B was released in EVs within minutes after stimulation of HeLa cells or red blood cells, detected by live cell imaging and flow cytometry. In the presence of Retro-2.1, an inhibitor of intracellular retrograde trafficking, a continuous release of Stx-positive EVs occurred. EVs from HeLa cells possess the Gb3 receptor on their membrane, and EVs from cells that were treated with a glycosylceramide synthase inhibitor, to reduce Gb3, bound significantly less Stx1B. Stx1B was detected both on the membrane and within the shed EVs. Stx1B was incubated with EVs derived from blood cells, in the absence of cells, and was shown to bind to, and be taken up by, these EVs, as demonstrated by electron microscopy. Using a membrane translocation assay we demonstrated that Stx1B was taken up by blood cell- and HeLa-derived EVs, an effect enhanced by chloropromazine or methyl-ß-cyclodextrin, suggesting toxin transfer within the membrane. This is a novel mechanism by which EVs derived from blood cells can sequester their toxic content, possibly to evade the host response.

## 1. Introduction

Shiga toxin (Stx) is a ribosome-inactivating AB5 protein consisting of one enzymatically active A-subunit and a pentameric B-subunit [1] which, upon binding to its receptor globotriaocylceramide (Gb3) [2], is taken up by endocytosis [3,4]. Once inside the cell, Stx undergoes retrograde transport, from the early endosome to the trans-Golgi network, and further to the endoplasmic reticulum and the cytosol. Translocation to the cytosol inactivates ribosomes by cleaving an adenine in 28S rRNA [5] leading to inhibition of protein synthesis and cell death [6]. Retro-2.1 is a synthetic compound that blocks the intracellular retrograde pathway of the toxin between early endosomes and the trans-Golgi network [7,8], thereby protecting the cell from the cytotoxic effect of the toxin [9].

Not all cells undergo cell death upon binding and uptake of Stx. Toxin binding to blood cells, including platelets, leukocytes, and red blood cells, can lead to their activation without inducing cytotoxicity [10]. Upon activation, an increased release of extracellular vesicles (EVs) occurs, and some of these contain the toxin [11,12,13]. Microvesicles (100–1000 nm) are a subtype of EVs that directly bud off the plasma membrane of cells [14,15]. Their content includes lipids [16], proteins [17], and RNA [18] reflecting the cell of origin. EVs are a means of communication between cells, in proximity of each other or at a distance, by the transfer of cargo and membrane receptors [15], but their shedding can also be a mechanism by which cells rid themselves of harmful substances [19]. In Stx-associated disease, microvesicles derived from blood cells bearing the toxin were shown to transport the toxin into the kidney [11].

Stx is the main virulence factor of enterohemorrhagic *Escherichia coli* (EHEC) and is subdivided into two main forms, Stx1 and Stx2 [20]. EHEC can cause gastrointestinal infection in humans manifesting with diarrhea or hemorrhagic colitis and, in severe cases, the life-threatening complication, hemolytic uremic syndrome (HUS). HUS is characterized by thrombocytopenia, hemolytic anemia, and acute renal failure [21]. EHEC strains are non-invasive [22] and remain in the gut after colonization. During infection, Stx can gain access to the blood circulation. However, only minimal levels of free Stx have been detected in the bloodstream [23,24]. The toxin can bind to and be taken up by blood cells possessing the globotriaosylceramide (Gb3) receptor [25], and then be released from these cells within vesicles that shed off the cell surface [21]. Stx was detected in blood cell-derived microvesicles during HUS [11]. These toxin-positive blood cell-derived microvesicles were detected in the kidney of a patient with HUS and in mice infected with *E. coli* O157:H7 [11]. The interaction between Stx and blood cell-derived microvesicles can, thereby, explain how the toxin reaches the kidney and causes kidney failure in HUS patients.

In this study, we investigated the mechanism by which Stx interacts with EVs. We examined whether release of the toxin within vesicles budding off from cells occurs instantaneously or if it requires intracellular retrograde transport of the toxin. We investigated whether toxin-containing EVs were solely the result of vesicles shed from the cells incubated with the toxin, or if the toxin could bind directly to EVs, even in the absence of cells. Furthermore, we examined the localization of the toxin, on the inside or outside of the vesicle membrane, and if the toxin, after receptor binding, could transfer from the outside to the inside of a vesicle. We used the B-subunit of Stx1, lacking enzymatic cytotoxic activity, to address the role of membrane binding and uptake. This study provides novel insight into how proteins interact with EVs and transfer within the vesicle membrane, and could particularly explain the means by which EVs in the circulation enable exposure of Stx or its sequestration.

## 2. Results

### 2.1. Shiga Toxin 1B that Is Rapidly Shed in Extracellular Vesicles Does Not Undergo Retrograde Transport

**Figure 1:**
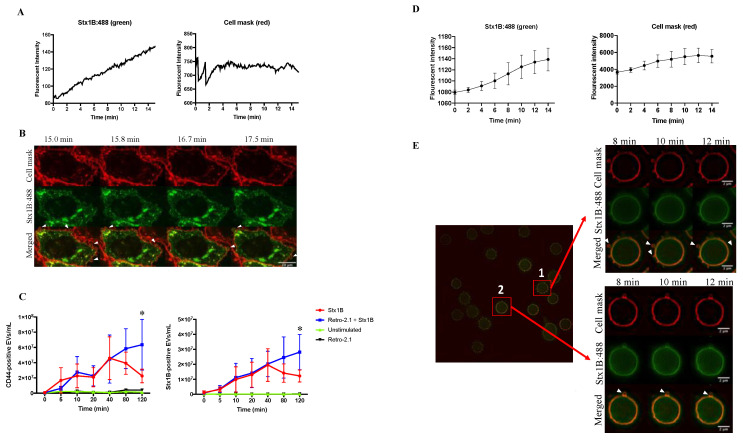
Shiga toxin 1B is rapidly shed in vesicles, before retrograde transport occurs. (**A**) HeLa cells were examined by confocal live cell imaging after addition of Stx1B:488 (60×). The fluorescent intensity of Stx1B (left panel) was increased in HeLa cells from time zero up to 15 min after Stx1B was added to the cell medium. The fluorescent intensity of the cell mask (right panel) remained stable during the 15 min of imaging; (**B**) One representative HeLa cell was examined by confocal live cell imaging, 15 min after addition of Stx1B:488. Shed blebs containing Stx1B appeared on the surface of the cell (arrowheads) at various time points. Images were taken from Supplementary Video S2. Experiments were repeated three times with similar results; (**C**) Flow cytometry analysis of extracellular vesicles (EVs) released from HeLa cells. CD44-positive EVs depict the total number of EVs in the HeLa cell culture medium (left panel). Cells stimulated with Stx1B and treated with Retro-2.1 released more EVs than unstimulated cells. CD44-positive EVs that were also Stx1B-positive are shown in the right panel. HeLa cells stimulated with Stx1B and treated with Retro-2.1 released more toxin-positive EVs than unstimulated cells. At 120 min more EVs were shed from cells stimulated with Stx1B and treated with Retro-2.1 than those not treated with Retro-2.1, *: *p* < 0.05. Data represent the median and range of three separate experiments, each carried out in triplicate; (**D**) RBCs were pre-labeled with cell mask and images were taken by super illumination microscopy every other minute for 12 min. The fluorescent intensity of Stx1B (left panel) increased from time zero up to 14 min, whereas the fluorescent intensity of the cell mask (right panel) remained stable. Data represent the mean values and standard deviation of 6 RBCs; (**E**) Stx1B was detected by super illumination microscopy in blebs on the surface of the two RBCs marked as 1 and 2 (arrowheads) at various time points. Scale bar: 2 µm. Experiments were repeated three times with similar results.

Shedding of vesicles in the presence of Stx1B was studied in HeLa cells. After incubation with Stx1B, HeLa cells took up the toxin within minutes, as demonstrated in Figure 1A, left panel and in Supplementary Video S1A. The fluorescent intensity of Stx1B increased (Figure 1A, left panel) while the fluorescent intensity of the cell mask remained stable (Figure 1A, right panel). Live cell imaging captured images every 15 s and demonstrated that HeLa cells shed toxin-positive vesicles within 15 min of incubation with the toxin (Figure 1B and Supplementary Videos S2 and S3). Cells that were not incubated with the toxin did not exhibit the same extent of blebbing (Supplementary Video S2).

The number of EVs released from cells at different time points during Stx1B stimulation was further analyzed by flow cytometry (Figure 1C). The results showed that Stx1B is continuously released in EVs from HeLa cells already within 5 min after incubation with the toxin. CD44 was used as a cell marker for EVs shed from HeLa cells because it is ubiquitously expressed on the surface of these cells. The number of CD44-positive EVs, representing the total number of released EVs, continuously increased from time zero after stimulation with Stx1B. After 40 min, the total number of CD44-positive EVs (Figure 1C, left panel) and of those containing Stx1B (labeled for both CD44 and Stx1B, Figure 1C, right panel) were significantly higher in the cell medium from Stx1B-stimulated cells (Figure 1C, red line) as compared with the unstimulated cells (Figure 1C, green line, *p* < 0.05).

In order to determine if Stx1B release in EVs requires intracellular retrograde transport of the toxin, cells were incubated with Retro-2.1, a substance that inhibits transport of Stx from early endosomes to the trans-Golgi network [7,9]. Cells stimulated with Stx1B and treated with Retro-2.1 released significantly more EVs than unstimulated cells (Figure 1C, left panel, at 40 min, *p* < 0.05, at 80 min *p* < 0.01, and at 120 min *p* < 0.001). CD44-positive EVs that were also Stx1B-positive are depicted (Figure 1C, right panel). HeLa cells stimulated with Stx1B and treated with Retro-2.1 released significantly more toxin-positive EVs than unstimulated cells (at 40 min, *p* < 0.01, at 80 min *p* < 0.01 and at 120 min *p* < 0.001). At 120 min after Stx1B stimulation, cells exposed to Retro-2.1 exhibited a significantly higher number of total shed EVs (Figure 1C, left panel, blue line), and those containing Stx1B (Figure 1C, right panel, blue line), compared to cells that were not treated with Retro-2.1 (Figure 1C, left and right panels, red line, *p* < 0.05, Figure 1C) which, on the contrary, exhibited a decrease in EV release after 40 min. 

Stx uptake was also observed in red blood cells (RBCs) that took up Stx1B within minutes, as shown in Figure 1D, left panel using live cell imaging by super illumination microscopy. The fluorescent intensity of Stx1B (Figure 1D, left panel) was increased from time zero up to 14 min in six different RBCs (Figure 1D), whereas the fluorescent intensity of the cell mask (Figure 1D, right panel) remained rather stable. Rapid shedding of Stx1B within EVs was noted in RBCs (Figure 1E). Live cell imaging of RBCs showed uptake of Stx1B and toxin-positive vesicles on the surface of the cells within minutes (Figure 1E). Shedding could be visualized simultaneously at various areas of the cell membrane (Cell 1 in Figure 1E). Variations in depiction of EV shedding depended on the focal plane of the cell in focus.

### 2.2. Shiga Toxin 1B Is Located on the Membrane and Inside Extracellular Vesicles Released by HeLa Cells

**Figure 2:**
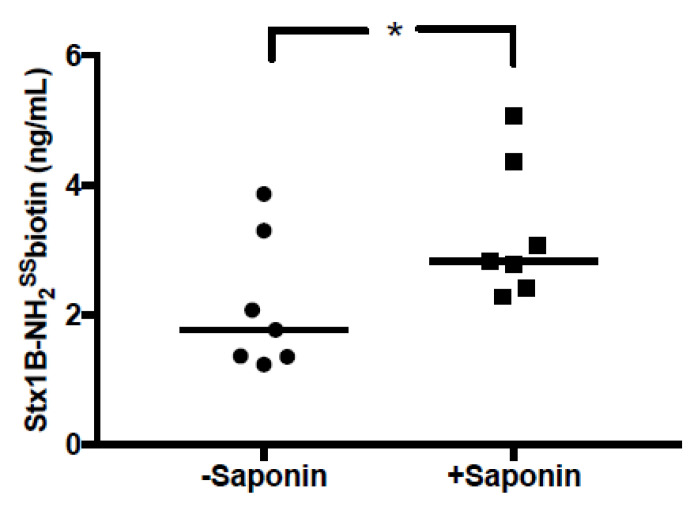
Shiga toxin 1B was detected on the outside and inside of extracellular vesicles released by HeLa cells. The toxin levels were assayed in extracellular vesicles released from Stx1B-stimulated HeLa cells by ELISA. The toxin levels in extracellular vesicles that were not permeabilized (–saponin) represent the toxin present on the exterior of the extracellular vesicles. The toxin levels in extracellular vesicles that were permeabilized with saponin (+saponin) represent the total amount of Stx1B on both the inside and outside of the extracellular vesicles. *: *p* < 0.05.

HeLa cells were stimulated with Stx1B and the amount of Stx1B released in EVs was determined by a Stx-ELISA. EVs were either treated with the permeability-inducing agent saponin to detect the total amount of Stx1B, located on the inside and outside of EVs, or not treated with saponin to determine the Stx1B exposed on the exterior. Stx1B released in EVs from HeLa cells was demonstrated on the exterior of EVs, as well as within the EVs (Figure 2).

### 2.3. Extracellular Vesicles Possess Gb3

**Figure 3:**
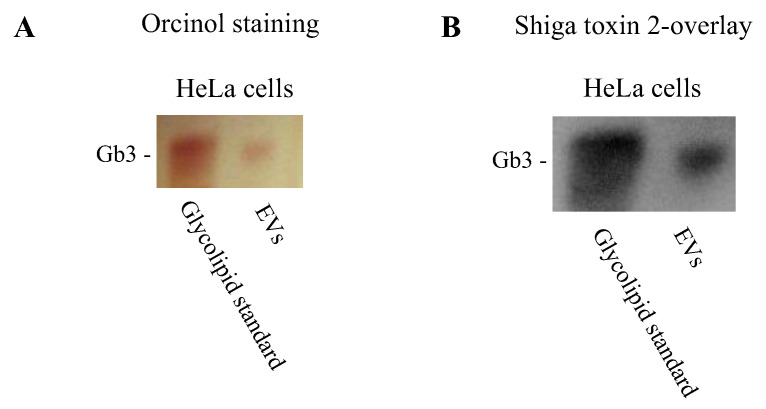
Extracellular vesicles from HeLa cells possess the Gb3 receptor. Lipids were extracted from isolated extracellular vesicles (EVs) derived from HeLa cells and analyzed by thin layer chromatography. A glycosphingolipid standard was used to identify the Gb3 band. (**A**) Gb3 was visualized by orcinol staining; (**B**) Shiga toxin 2 overlay to detect Shiga toxin binding to extracted Gb3.

A prerequisite for Stx binding to the outer surface of EVs is the presence of its glycolipid receptor, globotriaosylceramide or Gb3. Gb3 was detected in EVs from HeLa cells by thin layer chromatography (Figure 3). Gb3 was visualized by orcinol staining (Figure 3A) and by Shiga toxin overlay (Figure 3B). Shiga toxin overlay showed binding of the toxin to the Gb3 extracted from the HeLa-derived EVs. Gb3 on blood cell-derived EVs has been previously shown [26].

### 2.4. Shiga Toxin Binds to HeLa-Cell Derived EVs via the Gb3 Receptor

**Figure 4:**
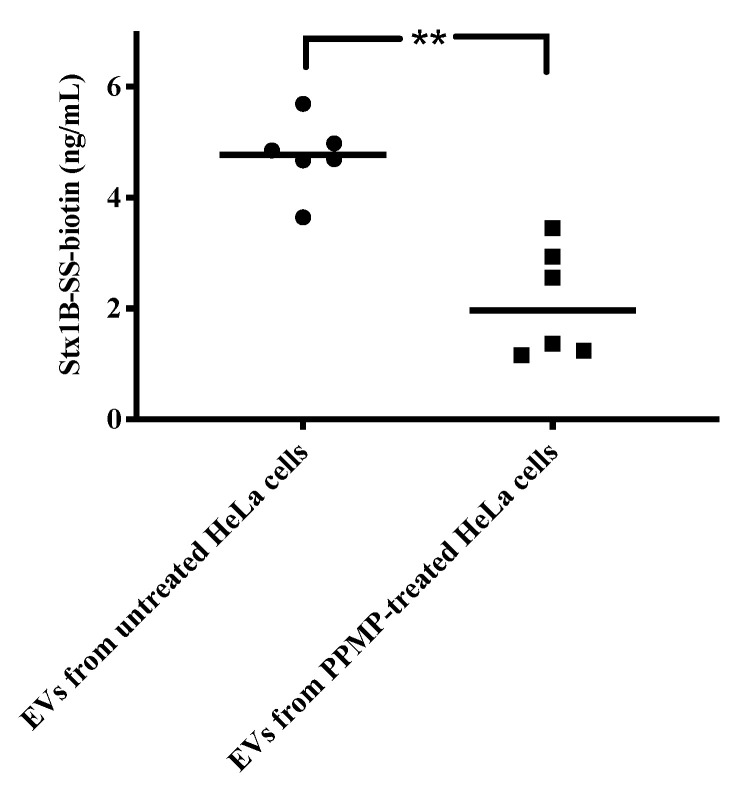
Gb3 content of extracellular vesicles affects Shiga toxin binding. Extracellular vesicles (EVs) derived from HeLa cells treated with PPMP or left untreated were incubated with Stx1B and the level of Stx1B was assayed by ELISA. ** *p* < 0.01.

To examine if Stx1B binds to HeLa-derived EVs in the absence of cells and the importance of the Gb3 receptor, HeLa cells were treated with D-threo-1-Phenyl-2-palmitoylamino-3-morpho lino-1-propanol (PPMP), a glycosylceramide synthase inhibitor or left untreated for 10 days. EVs were isolated, quantified and incubated with Stx1B 1 μg/mL for 1 h. In the presence of PPMP, a reduction of the expression of Gb3 has been previously shown [27], and this was confirmed by our group [26]. EVs from PPMP-treated cells incubated with Stx1B exhibited significantly reduced Stx1B binding as compared with EVs from HeLa cells that were not PPMP-treated (Figure 4).

### 2.5. Shiga Toxin 1B Interacts with Blood Cell-Derived Extracellular Vesicles in the Absence of Cells

**Figure 5:**
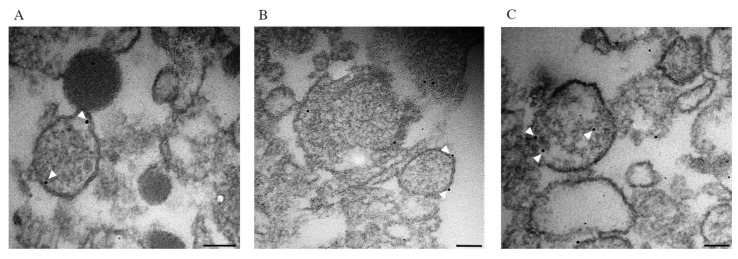
Shiga toxin visualization in extracellular vesicles by electron microscopy. Blood cell-derived extracellular vesicles were incubated with Stx1B conjugated to nanogold and sections of pelleted extracellular vesicles were analyzed by transmission electron microscopy. (**A**) Stx1B was visualized on the inner side of the vesicle membrane (arrowheads); (**B**) Stx1B was visualized on the outer side of the extracellular vesicle membrane (arrowheads); (**C**) Stx1B was visualized within an extracellular vesicle (arrowheads). Scale bar 100 nm.

The ability of Stx to interact with EVs in the absence of cells was further examined by transmission electron microscopy using blood cell-derived EVs that were incubated for 1h with Stx1B conjugated with nanogold. Sections of the blood cell-derived EVs, which had been incubated with Stx1B, demonstrated that Stx1B localized on the vesicle membrane surface (Figure 5A,B), as well as on the luminal side of the membrane and the inside of the EVs (Figure 5C).

### 2.6. Shiga Toxin Can Be Translocated into Extracellular Vesicles

**Figure 6:**
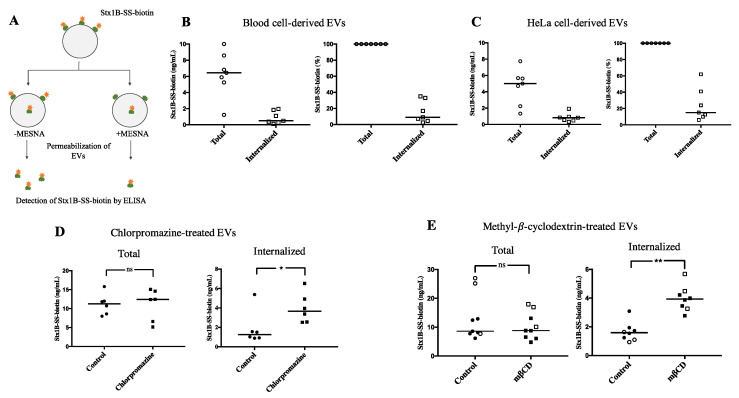
Membrane translocation of Shiga toxin 1B in extracellular vesicles (EVs). (**A**) A schematic presentation of the membrane translocation assay used to detect the total amount of biotinylated Stx1B in EVs (without exposure to membrane-impermeant MESNA), or the biotinylated Stx1B within EVs (after exposure to MESNA, to reduce cleavable biotin on the outer membrane of EVs before permeabilization). The amount of Stx1B-SS-biotin in the samples was detected by ELISA; (**B**,**C**) The total amount of Stx1B (on the outer and inner side of EVs) and the amount of translocated Stx1B were examined in (**B**) blood cell-derived or (**C**) HeLa-cell derived EVs. The right graph represents the percentage of Stx1B translocation as compared with the total. The data show that a portion of the total amount of Stx1B was translocated in both blood cell- and HeLa cell-derived EVs; (**D**,**E**) HeLa cell-derived EVs treated with (**D**) chlorpromazine or (**E**) methyl-β-cyclodextrin (mβCD). The total amount of Stx1B (left) and the amount of translocated Stx1B (right) were examined in treated EVs as compared with the control vesicles analyzed using the same experimental set-up. * *p* < 0.05 and ** *p* < 0.01; ns, not significant. Unfilled icons represent experiments in which native Stx1B-SS-biotin was used, whereas filled icons represent experiments using synthetic Stx1B-SS-biotin. The data present two technical replicates.

The finding that Stx1B, incubated with EVs derived from blood cells, was bound to the outer membrane, but could also be found within the EVs, suggested that the toxin was transferred into the vesicles. Experiments were designed to address Stx translocation into EVs in the absence of cells by using biotinylated Stx1B. The membrane-impermeable compound sodium 2-mercaptoethane sulfonate (MESNA) was used to remove biotin specifically on Stx1B that was found on the outer surface of EVs, and not on Stx1B that translocated inside the lumen of the EVs. After applying MESNA, vesicles were permeabilized. We could, thereby, use this method to detect total toxin content (–MESNA) or translocated toxin within the EVs (+MESNA), as illustrated in Figure 6A. A fraction of the total amount of biotinylated Stx1B was shown to be translocated by blood cell-derived EVs (*n* = 7, Figure 6B) and HeLa cell-derived EVs (*n* = 7, Figure 6C). The mechanism of Stx1B membrane translocation was further examined by treating HeLa cell-derived EVs with chlorpromazine or methyl-β-cyclodextrin (mβCD). Both substances increased the amount of translocated Stx1B, whereas the total amount of toxin (both on the inside and outside of EVs) remained the same as for the EVs treated with control vehicle (Figure 6D,E). In the experiments shown in Figure 6D,E, both native and synthetic Stx1B-SS-biotin were used exhibiting similar results. As a negative control, EVs were fixed with paraformaldehyde (PFA) before incubation with Stx1B-SS-biotin. EVs treated with PFA did not exhibit membrane translocation of Stx1B, as expected (data not shown).

## 3. Discussion

Shiga toxin circulates within blood cell-derived microvesicles during EHEC-associated HUS, and is thereby taken up within microvesicles by target organ cells, such as the kidney. Here, we investigated the mechanism by which Stx interacts with shed EVs, derived from HeLa cells and blood cells. The study specifically focused on the effect of the Gb3 receptor binding B-subunit of Stx in the absence of the cytotoxic A-subunit. The EVs where shown to possess the Gb3 receptor enabling specific binding and, when Gb3 exposure was reduced, Stx binding to EVs decreased. The toxin was rapidly released from HeLa cells in EVs, without undergoing retrograde transport to the ribosomes. Furthermore, the toxin bound directly to blood cell- and HeLa cell-derived EVs, in the absence of cells. The toxin was partly translocated in the membrane of vesicles derived from these cells, and thereby localized within the vesicles. This study provides novel insights into EV pathophysiology, particularly in Stx-mediated disease, demonstrating that EVs can be shed to rid the cell of accumulating unwanted protein, and that the toxin can be transferred across the EV membrane in a manner that either exposures it to the immune response and elimination, or its evasion of the immune response, allowing uptake of the toxin-positive EVs in target organ cells ultimately leading to a cytotoxic effect.

Stx retrograde trafficking to the Golgi occurs en route to ribosomal inactivation. Stx1B reaches the Golgi in HeLa cells within 40 min [28]. We found that Stx1B was shed in EVs much earlier, which suggested that release of the toxin within vesicles occurred before retrograde transport could occur. This finding was supported by treatment with Retro-2.1, a compound that blocks intracellular trafficking of Stx from early endosomes to the Golgi [9]. In the presence of Retro-2.1, EVs were shed continuously, even after 40 min, whereas, in its absence, shedding of the toxin-positive EVs decreased at this time point, most probably because the remaining intracellular toxin had reached the Golgi. This finding suggested that when the toxin accumulated and could not undergo retrograde trafficking, the cell attempted to eliminate it by shedding off vesicles containing the toxin. The phenomenon of rapid EV shedding has been previously demonstrated for monocytes containing interleukin-1 beta [29].

Retro-1 and -2 compounds have been shown to protect cells from the cytotoxic effects of Stx, and the similar toxin ricin, by blocking intracellular trafficking [7,8,9]. We demonstrated an additional protective effect, as more toxin-positive EVs were shed from Retro-2.1-treated cells, thus sparing the cell from toxin accumulation. The number of EVs detected after Stx stimulation (Figure 1C) was a combined balance of vesicle release and uptake by the HeLa cells. It would appear that in the presence of Retro-2.1 more vesicles are shed, most probably to rid the cell of the bacterial protein. Importantly, the shed EVs could carry the toxin on the membrane or within the vesicle, presumably still bound to the Gb3 receptor, as we showed that the vesicles possessed the toxin receptor. The presence of the toxin on the exterior of the EV membranes will allow the host response to react and remove these circulating EVs. In this manner Retro-2.1 not only protects cells from cytotoxicity but can also contribute to the elimination of circulating toxin-positive EVs.

Stx1 was present both on the inside and outside of EVs shed from cells. As the budding process occurred within minutes, we assumed that some of the Stx1B was shed on the cell membrane before cellular uptake. Within 15–20 min, most of the toxin would be expected to be endocytosed [30], and thus the shed EVs would be expected to contain the toxin on their inside, along with contents originating from the parent cell [19]. However, a novel finding in the current study was that the toxin could transfer from the outside to the inside of EVs. To our knowledge, this form of intramembranous transfer of proteins in EVs has not been described before, although the molecular interactions on the surface of EVs have been the focus of many studies [31]. This finding suggests that Stx1B can transfer through the membrane and its presence on different sides of the membrane may not solely reflect the mechanism and timing of shedding.

The degree of membrane translocation of Stx in EVs was affected by both chlorpromazine and mβCD. Chlorpromazine, which is a well-known clathrin-dependent endocytosis inhibitor, increased the membrane translocation of Stx. The effect of chlorpromazine cannot be explained by its action as a endocytosis inhibitor [32]. However, chlorpromazine has also been shown to affect membrane permeability and fluidity [33] which could explain its effect on enhanced Stx1B translocation. Cholesterol extraction by mβCD has previously been shown to affect fluidity in biological membranes [34]. This could explain the increase in Stx translocation after mβCD treatment of EVs. In line with this, an earlier study has shown endosomal escape of Stx after treatment with mβCD [35], indicating membrane penetration of Stx after cholesterol extraction.

We have previously shown that Stx2 within blood cell-derived microvesicles is taken up by renal cells. Once within the renal cell, Stx2 is released from the microvesicles, and thereafter undergoes retrograde transport to the ribosomes [11]. Previous studies have also shown that the Gb3 receptor directed Stx into retrograde trafficking [36,37], thereby reaching and depurinating ribosomes. Our present and previous findings that EVs, derived from blood cells [26] and HeLa cells, possess the Gb3 receptor, would indicate that the toxin binds to its receptor on the EV membrane and is transported on or within microvesicles, presumably still bound to the receptor. Once the toxin-positive EVs are taken up in target organ cells, the Gb3 present on the membranes of the recipient cell direct the toxin towards retrograde trafficking [26].

Bacterial outer membrane vesicles containing Stx can be taken up by intestinal cells, and thereby induce cytotoxicity [37]. After the intestinal injury and colitis have occurred, EHEC infection can lead to the severe complication HUS. Stx gains access to the circulation bound to microvesicles and is taken up by the kidney [11,21,25]. The findings presented here could have implications in human disease as we have shown that the toxin can be rapidly shed in or on EVs and transfer through the EV membrane. The capability of the toxin to transfer in and out of the EV membrane could enable its exposure to phagocytes, which we have shown readily engulf Stx-positive EVs [38]. Sequestration of the toxin within the EV can prevent this from occurring and allow the toxin to circulate undetected, hidden by the host membrane. Toxin-positive EVs would be taken up by kidney cells leading to toxin release and ultimately renal failure. Thus, toxin shedding within EVs or direct binding to EVs can be protective for the cells of origin but damaging to recipient cells. Future studies are needed to address the mechanism of toxin transfer through the EV membranes, as this could affect the course of disease by promoting the elimination or uptake of toxin-positive EVs.

## 4. Materials and Methods

### 4.1. HeLa Cell Culture and Inhibition of Gb3 Synthase

HeLa cells (from L. Johannes, Institute Curie, Paris, France) were cultured at 37 °C with 5% CO2 in Dulbecco’s modified Eagle’s high glucose medium (DMEM, HyClone, GE Healthcare Life Science, South Logan, UT, USA) supplemented with 10% fetal bovine serum (FBS, Life Technology, Carlsbad, CA, USA), and 1% penicillin-streptomycin. Cells were used at 80–90% confluence.

Certain HeLa cells were treated with D-threo-1-Phenyl-2-palmitoylamino -3-morpholino-1-propanol (PPMP, Abcam, Cambridge, UK) as previously described [26], in order to reduce Gb3 expression.

### 4.2. Blood Collection

Red blood cells (RBCs) from one anonymous donor with phenotype P_1_^k^ (Gb3-positive) were obtained from the cryopreserved rare reference material archives at the Department of Clinical Immunology and Transfusion Medicine, Lund, Sweden and isolated as described previously [13,39]. Venous blood from healthy volunteers (*n* = 6) was collected into 2.7 mL vacutainer tubes containing 0.5 mL 0.129 M sodium citrate (Becton Dickinson, Franklin Lakes, NJ, USA) through a butterfly needle (Terumo Medical Products Hangzhou CO, Hangzhou, China). Blood was used within 30 min of collection. The study was performed with the approval of the Ethical Review Board of Lund University and the informed written consent of the healthy volunteers. With regard to the sample from the anonymous donor, the provision complies with national regulations regarding the use of superfluous material from blood donations for which the donor origin cannot be traced and written consent was obtained at the time of donation.

### 4.3. Shiga Toxin 1B

Stx1B-subunit conjugated to Alexa-488 or nanogold (1.4 nm) and Stx1B-SS-biotin, were all previously described [4,40]. In certain translocation experiments, a synthetic Stx1B with an azide containing amino acid, conjugated to biotin with DBCO-SS-PEG3-Biotin (Tebu-Bio, Paris, France) was used. The synthetic Stx1B (patent pending) was chemically generated by solid phase peptide synthesis and refolded into functional protein that was shown to undergo intracellular retrograde transport, tested by immunofluorescence, as previously described [28]. Stx2 was obtained from Phoenix Lab, Tufts Medical Center, Boston, MA, USA. Stx1B and Stx2 were used in various experimental settings, including cell incubations, as described in Table 1.

### 4.4. Detection of Shed Vesicles from HeLa Cells by Confocal Immunofluorescence Microscopy

HeLa cells 30,000 cells/well were cultured in FluoroBrite DMEM supplemented with 10% FBS and grown to 80% confluence in 8-well chamber slides (Ibidi, Planegg, Germany), 24 h before experiments. Cells were labeled with CellMask Deep Red stain (1:3000, Invitrogen, Carlsbad, CA, USA), washed with PBS, and imaged in FluoroBrite DMEM without supplements. Stx1B:Alexa-488 (1 µg/mL, this concentration was optimized to visualize Stx in shed EVs) was added to the cells during live cell imaging performed using a Nikon A1+ confocal microscope (Nikon instruments Inc., Tokyo Japan), 60× Apo DIC oil immersion objective with a numerical aperture of 1.40.

### 4.5. Isolation of Extracellular Vesicles from HeLa Cells

Cell medium from untreated or PPMP-treated cells was centrifuged at 800× *g* for 10 min followed by 10,000× *g* for 10 min to remove cell debris. The supernatant was centrifuged for an additional 40 min at 20,000× *g* resulting in an EV-rich suspension followed by a washing step with phosphate buffered saline (PBS, Hyclone, GE Life Sciences, Chicago, IL, USA). The cell medium and PBS were filtered (0.2 µm) before addition to cells or washing of EVs, respectively. In all experiments, EVs were used immediately after centrifugation. For experiments with and without PPMP EVs were quantified using a NanoSight LM10 instrument (NanoSight, Amesbury, UK).

For thin layer chromatography experiments, EVs were instead isolated by ultracentrifugation (using a type 70.1 Ti fixed-angled rotor, Backman Coulter, Brea, CA, USA) at 110,000× *g* for 1.5 h to obtain an EV-rich suspension with detectible levels of Gb3. This EV suspension was stored at −80 °C before used.

### 4.6. Treatment of Shiga Toxin 1B-Stimulated HeLa Cells with Retro-2.1 and Analysis of Extracellular Vesicles by Flow Cytometry

HeLa cells were grown to 80% confluence in 6-well Nunc culture plates (Thermo Fisher Scientific, Rockford, IL, USA), for 24 h before experiments. The cells were washed and certain cells were incubated with Retro-2.1 (1 µM), to block retrograde transport of Stx before reaching the Golgi [7,9], for 1 h at 37 °C, before addition of Stx1B:Alexa-488 (10 ng/mL, this concentration was optimized to detect EVs by flow cytometry) diluted in DMEM supplemented with 0.5% exosome-depleted FBS (Gibco, Grand Island, NY, USA) for 0–120 min at 37 °C. EVs were isolated from the cell medium as described above followed by labeling with mouse anti-human CD44:PE (1:320, eBioscience, Thermo Fisher Scientific) for 30 min at rt. EVs were washed with PBS by centrifugation at 20,000× *g* for 40 min, after which the EV suspension was diluted in PBS before analysis by flow cytometry using a CyFlow Cube 8 flow cytometer (Partec, Görlitz, Germany) and FCS Express 4 Flow Research Edition software (De Novo Software, Glendale, CA, USA). Forward and side scatter measurements were attained with gain settings in logarithmic mode and flow rate was set to 0.2 μL/sec. Results are expressed as the number of positive EVs/mL medium.

### 4.7. Detection of Shed Vesicles on Red Blood Cells by Super Illumination Microscopy

RBCs were labeled with CellMask deep red stain (1:3000) for 15 min at rt and washed with PBS by centrifugation at 800× *g* for 10 min. Pelleted RBCs 4 × 10^5^ cells/well were dissolved in FluoroBrite DMEM and added to a final volume of 300 µL/well in 8-well chamber slides. Stx1B:Alexa-488 (10 ng/mL, this concentration was optimized to achieve a low fluorescent background and a detectible Stx1B signal) was added to the cell medium during live cell imaging. Cells were imaged in a Nikon Ti-E microscope (×100 oil objective) equipped with N-SIM E (Nikon Instruments Inc., Tokyo, Japan) and a Hamamatsu Flash 4 camera (Hamamatsu photonics, Shizuoka, Japan) using NIS elements AR software V.5.11.01 (Nikon Instruments Inc.).

### 4.8. Detection of Shiga Toxin 1B by ELISA

EVs were isolated (as described above) from a T175 culture flask of HeLa cells that were stimulated with Stx1B-SS-biotin (200 ng/mL) for 40 min. PPMP-treated or untreated HeLa cells were stimulated with calcium ionophore (A23187, Sigma-Aldrich, St. Louis, Missouri, USA) 10 µM for 40 min at 37 °C and EVs were likewise isolated from the cell medium. Washed EVs were incubated with Stx1B-SS-biotin (1 µg/mL) for 1 h.

After stimulation, EVs were washed four times with PBS and EV samples were diluted in 1% BSA with or without 0.5% saponin (in order to lyse the EVs, Sigma-Aldrich). A white 96-well plate (Nunc Maxisorp, Thermo Fisher Scientific) was coated with mouse anti-Stx1B antibody (1:1000, 3C10, Toxin Technology, Sarasota, FL, USA) in carbonate buffer, pH 9.6, at 4 °C overnight. The plate was washed with PBS-tween and blocked in 1% bovine serum albumin (BSA, Sigma-Aldrich) for 1 h. The EV samples were added at a final volume of 50 µL/well and incubated for 1 h, at rt. Wells were washed, incubated with Streptavidin-POD conjugate (1:10,000, Roche Diagnostics GmbH, Mannheim, Germany) for 45 min, and detected by supersignal Pico chemoluminescent substrate (Thermo Fisher Scientific) in a plate reader.

### 4.9. Thin Layer Chromatography for Detection of Gb3 on Extracellular Vesicles

The glycosphingolipid content of HeLa-derived EVs described above was analyzed by a modified method of thin layer chromatography (TLC) [41]. Briefly, EVs derived from two T175 culture flasks (Nunc EasYFlask, Thermo Fisher Scientific) of calcium ionophore-stimulated HeLa cells, isolated by ultracentrifugation as described above, were dissolved in water/methanol (1:2), sonicated for 30 s, and incubated for 1 h at 65 °C. Cell debris were removed by centrifugation and lipids were air dried under a stream of nitrogen prior to mild saponification followed by Folch partition (2:1:0.6 chloroform/methanol/water). Lipids were loaded onto a water-resistant TLC silica gel 60 (Merck Millipore, Darmstadt, Germany) together with a neutral glycosphingolipid mixture (Matreya LLC, State Collage, PA, USA) and separated by chloroform/methanol/water (6.5:3:0.8). Separated lipids were visualized by orcinol staining or by Stx overlay. Stx overlay was obtained by immersing the TLC plate with P28 (Poly(ethyl methacrylate)) followed by blocking in 1% BSA for 1 h and incubation with Stx2 (200 ng/mL) for an additional 1 h. The plate was washed with tris-buffered saline (TBS), incubated with mouse anti-Stx2 antibody (1 µg/mL, 11E10, Hycult Biotech, Uden, The Netherlands) for 1 h before washing, and incubating with the secondary antibody (anti-mouse HRP, 1:1000, Dako, Glostrup, Denmark). Bands were visualized by Pierce ECL plus immunoblotting substrate (Thermo Fisher Scientific). All chemicals were purchased from Sigma-Aldrich unless stated otherwise. The presence of Gb3 on blood cell-derived EVs has been previously described [26].

### 4.10. Stimulation of Whole Blood and Isolation of Blood Cell-Derived Extracellular Vesicles

Whole blood was diluted 1:1 with DMEM and stimulated with 10 µM calcium ionophore for 40 min at 37 °C. Blood cells were pelleted by centrifugation at 1500× *g* for 15 min followed by 10,000× *g* for 10 min to discard cell debris. The EV-rich suspension was obtained by centrifugation at 20,000× *g* for 30 min and used for further analysis as described below. EVs were used immediately after centrifugation.

### 4.11. Transmission Electron Microscopy

EVs isolated from calcium ionophore-stimulated whole blood were incubated with Stx1B/nanogold (200 ng/mL) for 1 h, at 37 °C, washed twice with PBS, fixed in 1% paraformaldehyde (PFA) and 2% glutaraldehyde (Sigma-Aldrich) diluted in 0.1 M Sörensen buffer (all chemicals from VWR, Radnor, PA, USA) for 1 h, followed by 1% osmium (Ted Pella Inc, Redding, CA, USA), and added to a 1.5 mL tube preloaded with 2% low melting agarose. EVs were pelleted into the agarose gel by centrifugation for 1 h, at 20,000× *g*. The supernatant was discarded, and 4% agarose was added onto the pellet and incubated for 1 h, at 4 °C, followed by dehydration in an increasing concentration of ethanol and embedding in polybed (Polyscience, Warrington, PA, USA). Ultra-thin sections (60 nm) were obtained using Leica EM UC7 and mounted onto hexagonal gold grids. The specimen was treated with gold enhancement EM (Nanoprobes, Yaphank, NY, USA) according to the manufacturer’s instructions, followed by incubation with 2% uranyl acetate at 40 °C for 15 min. Grids were imaged in a FEI Tecnai Biotwin 120 kv electron microscope (Hillsboro, OR, USA).

### 4.12. Membrane Translocation of Shiga Toxin

Experiments were designed to detect the translocation of Stx1B from the outside to the inside of EVs. EVs shed from whole blood or HeLa cells were used. Whole blood (14 mL) was stimulated with calcium ionophore and EVs were concentrated to an EV suspension of 1 mL (as described above). The EV suspension was incubated with 1 µg/mL Stx1B-SS-biotin at 37 °C, for 1 h. This concentration of Stx1B was chosen in order to optimize binding.

HeLa cells were cultured to confluence in T175 culture flasks. The cells were stimulated with calcium ionophore (10 µM) and EVs were concentrated to 1 mL EV suspension, as described above. EVs were either left untreated, treated with chlorpromazine (15 µg/mL for 1 h), methyl-β-cyclodextrin (mβCD, 5 mM, for 30 min), or fixed with 1% PFA followed by quenching with 50 mM NH_3_Cl for 15 min. Chlorpromazine is an inhibitor of clathrin-mediated endocytosis [32] and mβCD extracts cholesterol from the plasma membrane [34]. The control vehicle was water for both chlorpromazine and mβCD. After treatment with chlorpromazine for 1 h, Stx1B-SS-biotin (1 µg/mL) was immediately added, whereas for mβCD and PFA, HeLa-derived EVs were washed with OptiMEM once before addition of Stx1B-SS-biotin. An EV control sample was treated in the same way but without chlorpromazine or mβCD.

After incubation with Stx1B-SS-biotin, EVs from each cell source (blood cells or HeLa cells) and treatment were divided into two tubes, washed twice with PBS, after which one part was incubated with the impermeable reducing agent sodium 2-mercaptoethanesulfonate (MESNA, Sigma-Aldrich) for 16 min, on ice, to remove biotin present on the EV exterior. After two additional washes, the EV samples were lysed with 0.5% saponin and analyzed by ELISA, as described above. The samples treated with MESNA corresponded to biotinylated Stx1B present solely within the EVs (after lysis), whereas samples not treated with MESNA depicted the total amount of Stx1B.

### 4.13. Statistics

Differences between EVs shed from Stx1B-stimulated and unstimulated HeLa cells measured by flow cytometry were assessed by the two-way ANOVA test. Differences regarding the amount of Stx1B-SS-biotin, on the exterior of EVs, or the total amount after lysis were determined using the Wilcoxon matched-pairs signed rank test. This test was also used to evaluate differences between the amount of translocated Stx1B-SS-biotin in EVs. Statistical analyses were performed using GraphPad prism software (GraphPad software, version 7, San Diego, CA, USA).

## Figures and Tables

**Table 1:** Shiga toxin used in experiments in this study.

Shiga Toxin	Conjugate	Concentration	Media	Cells	Experiment	Detection Method
Stx1B	Alexa-488	1 μg/mL	DMEM ^1^	HeLa	Incubation 0–30 min with cells	Live cell imaging
Stx1B	Alexa-488	10 ng/mL	DMEM ^1^	RBC ^2^	Incubation 0–15 min with RBC in suspension	Live cell imaging
Stx1B	Alexa-488	10 ng/mL	DMEM ^3^	HeLa	Incubation 0–120 min with cells ^4^	Flow cytometry
Stx1B	SS-biotin	200 ng/mL	OptiMEM	HeLa	Incubation with cells for 40 min ^4^	Stx1B-ELISA
Stx2	-	200 ng/mL		HeLa	Gb3 ^5^ overlay on extracted membrane lipids	Thin layer chromatography
Stx1B	Nanogold	200 ng/mL	DMEM	Blood	Extracellular vesicles incubated for 1 h ^4^	Electron microscopy
Stx1B ^6^	SS-biotin	1 µg/mL	OptiMEM or DMEM ^7^	HeLa and blood cells	Extracellular vesicles incubated for 1 h ^4^	ELISA

1, FluoroBrite DMEM; 2, (RBC) red blood cells; 3, supplemented with 0.5% exosome-free FBS; 4, extracellular vesicles were used immediately after isolation; 5, (Gb3) globotriaostylceramide, the Shiga toxin receptor; 6, in these membrane translocation experiments both native and synthetic Stx1B were used; 7, HeLa cells were stimulated in OptiMEM and blood cells were stimulated in DMEM.

## Supplementary Materials

The following are available online at https://www.mdpi.com/2072-6651/12/7/449/s1, Video S1: Confocal live cell imaging of Shiga toxin 1B uptake in HeLa cells. HeLa cells labeled with deep red cell mask (red labeling) were stimulated with Stx1B:488 (green labeling) and cells were examined from time zero after addition of Stx1B every 15 s for 15 min by confocal microscopy. (A) Certain HeLa cells stimulated with Stx1B took up toxin. (B) Cells that were not exposed to Stx1B were examined using the same settings and showed background labeling. Experiments using these settings were performed three times, results from one representative experiment are shown. Video S2: Confocal live cell imaging of cells after Shiga toxin 1B uptake. (A) Two HeLa cells positive for Stx1B:488, 15 min after addition of Stx1B (detected in Video S1A), were further examined at a higher resolution. Cells were imaged every 15 s for 15 min to visualize the shedding of bleb formations. (B) Two HeLa cells not stimulated with Stx1B (selected from Video S1B) were imaged using the same settings and shedding was not detected on the surface of the cells. Video S3: Bright field imaging of HeLa cells shedding extracellular vesicles. Bright field imaging of shed formations on the same two HeLa cells is shown in Video S2A. Cells were imaged 15 min after stimulation with Stx1B:488, every 15 s for 15 min.
