# Supplementary material for: Shiga Toxin Uptake and Sequestration in Extracellular Vesicles Is Mediated by Its B-Subunit
Source: Toxins (Basel). 2020 Jul 10;12(7):449. doi: 10.3390/toxins12070449 (PMC7404996; doi:10.3390/toxins12070449)
Supplement: Supplementary file 1 [file toxins-12-00449-s001.zip › toxins-841625-supplementary/toxins-841625 - supplementary.docx]

Supplementary Materials Shiga Toxin Uptake and Sequestration in Extracellular Vesicles Is Mediated by Its B-Subunit

Annie Willysson, Anne-lie Ståhl, Daniel Gillet, Julien Barbier Jean-Christophe Cintrat, Valérie Chambon, Anne Billet, Ludger Johannes and Diana Karpman

Videos

**Video S1**: Confocal live cell imaging of Shiga toxin 1B uptake in HeLa cells. HeLa cells labeled with deep red cell mask (red labeling) were stimulated with Stx1B:488 (green labeling) and cells were examined from time zero after addition of Stx1B every 15 sec for 15 min by confocal microscopy. (**A**) Certain HeLa cells stimulated with Stx1B took up toxin. (**B**) Cells that were not exposed to Stx1B were examined using the same settings and showed background labeling. Experiments using these settings were performed three times, results from one representative experiment are shown.

**Video S2:** Confocal live cell imaging of cells after Shiga toxin 1B uptake. (**A**) Two HeLa cells positive for Stx1B:488, 15 min after addition of Stx1B (detected in Video S1A), were further examined at a higher resolution. Cells were imaged every 15 sec for 15 min to visualize the shedding of bleb formations. (**B**) Two HeLa cells not stimulated with Stx1B (selected from Video S1B) were imaged using the same settings and shedding was not detected on the surface of the cells.

**Video S3:** Bright field imaging of HeLa cells shedding extracellular vesicles. Bright field imaging of shed formations on the same two HeLa cells is shown in Video S2A. Cells were imaged 15 min after stimulation with Stx1B:488, every 15 sec for 15 min.
