# Supplementary figures and images for: Shiga Toxin Uptake and Sequestration in Extracellular Vesicles Is Mediated by Its B-Subunit
Source: Toxins (Basel). 2020 Jul 10;12(7):449. doi: 10.3390/toxins12070449 (PMC7404996; doi:10.3390/toxins12070449)

## Slide 1
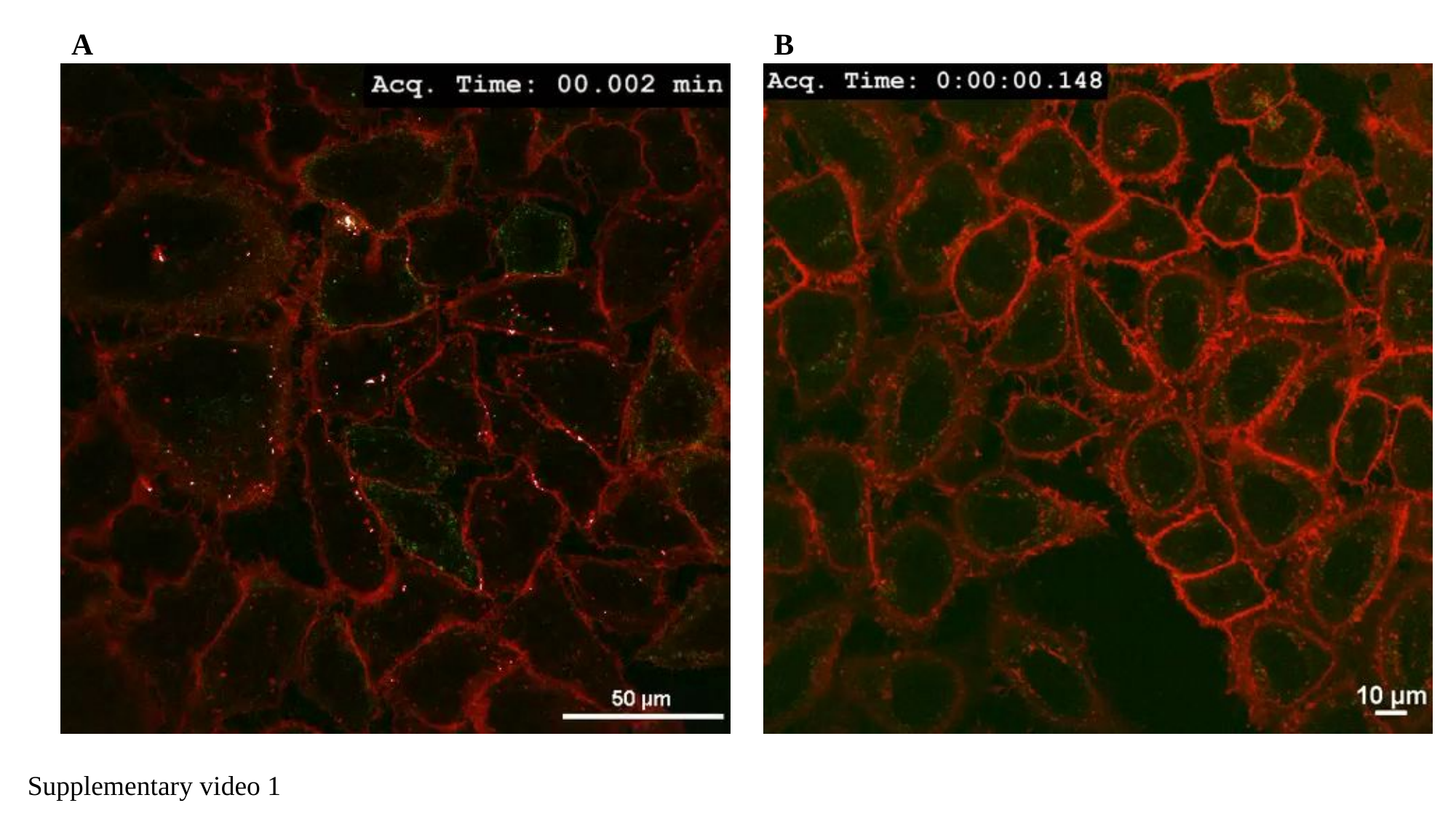

A
B
Supplementary video 1

Supplement: Supplementary file 1 [file toxins-12-00449-s001.zip › toxins-841625-supplementary/Supplementary video 1.pptx]

## Slide 1
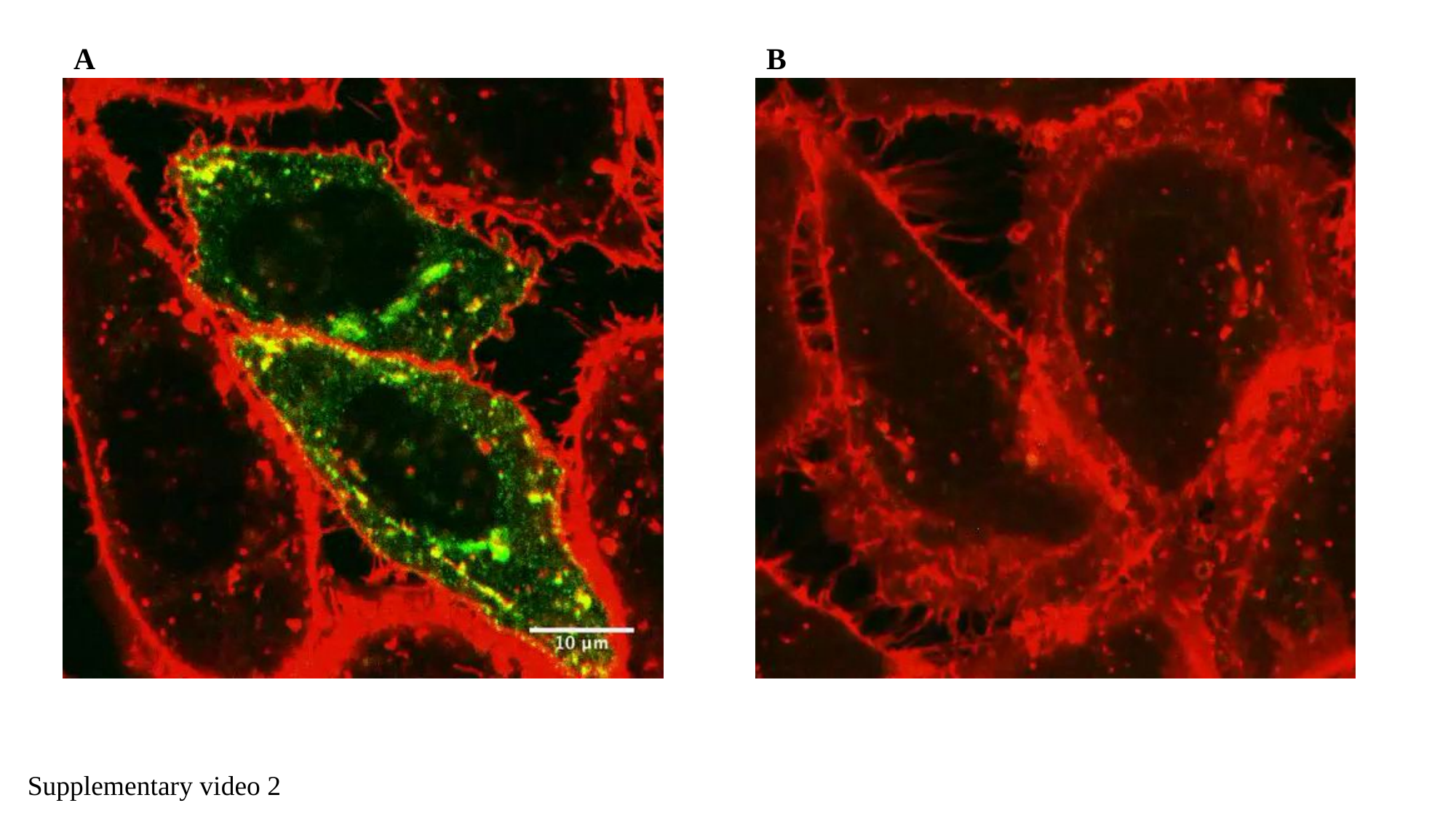

A
B
Supplementary video 2

Supplement: Supplementary file 1 [file toxins-12-00449-s001.zip › toxins-841625-supplementary/Supplementary video 2.pptx]

## Slide 1
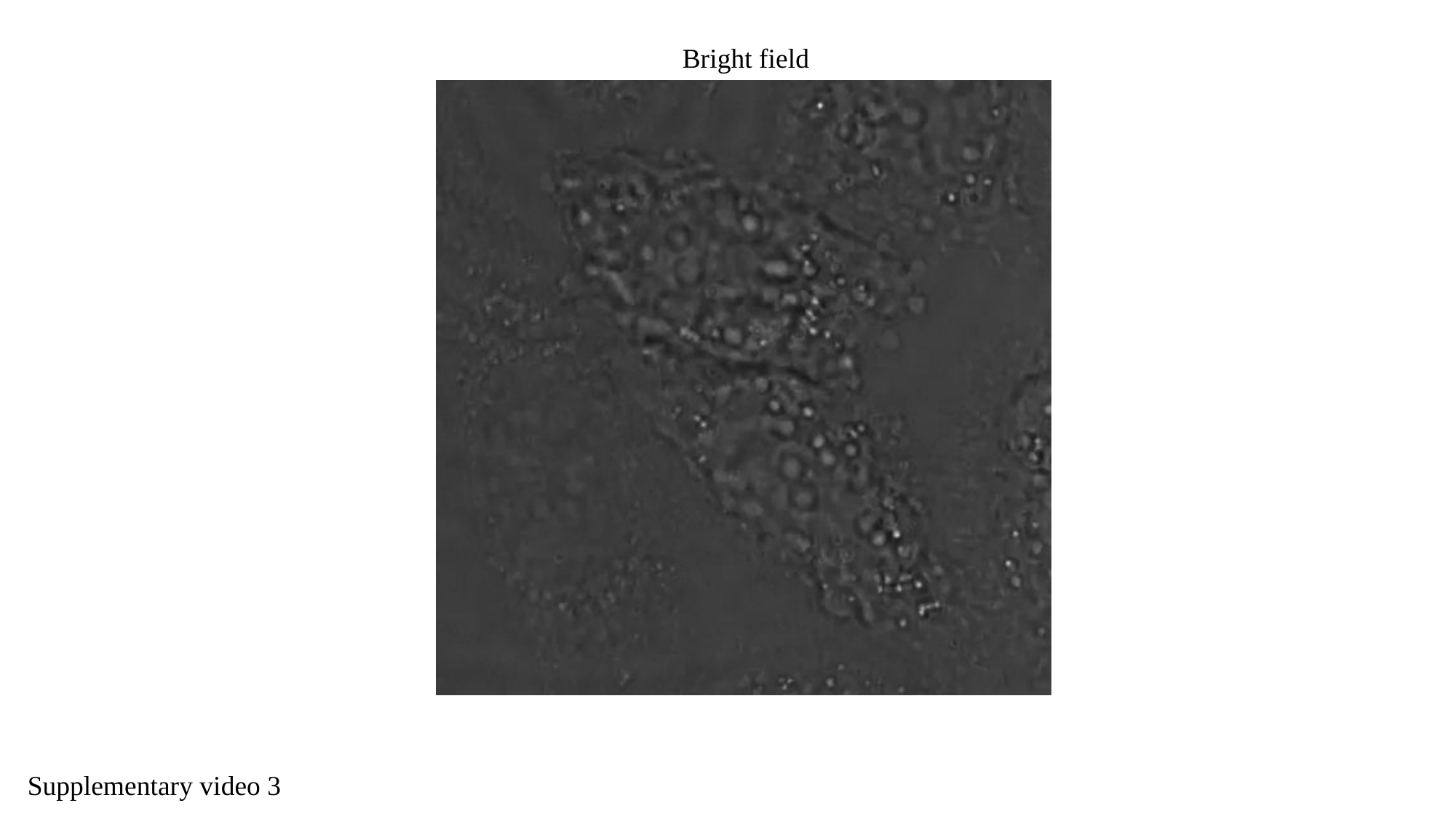

Bright field
Supplementary video 3

Supplement: Supplementary file 1 [file toxins-12-00449-s001.zip › toxins-841625-supplementary/Supplementary video 3.pptx]
